# The Relationship between Coenzyme Q10, Oxidative Stress, and Antioxidant Enzymes Activities and Coronary Artery Disease

**DOI:** 10.1100/2012/792756

**Published:** 2012-05-03

**Authors:** Bor-Jen Lee, Yi-Chin Lin, Yi-Chia Huang, Ya-Wen Ko, Simon Hsia, Ping-Ting Lin

**Affiliations:** ^1^School of Nutrition, Chung Shan Medical University, No. 110, Section 1, Jianguo N. Road, Taichung 40201, Taiwan; ^2^The Intensive Care Unit, Taichung Veterans General Hospital, No. 160, Section 3, Chung-Kang Road, Taichung 40705, Taiwan; ^3^Department of Nutrition, Chung Shan Medical University Hospital, No. 110, Secion 1, Jianguo N. Road, Taichung 40201, Taiwan; ^4^Department of Nutrition and Institute of Biomedical Nutrition, HungKuang University, No. 34 Chung-Chie Road, Sha Lu, Taichung 43346, Taiwan

## Abstract

A higher oxidative stress may contribute to the pathogenesis of coronary artery disease (CAD). The purpose of this study was to investigate the relationship between coenzyme Q10 concentration and lipid peroxidation, antioxidant enzymes activities and the risk of CAD. Patients who were identified by cardiac catheterization as having at least 50% stenosis of one major coronary artery were assigned to the case group (*n* = 51). The control group (*n* = 102) comprised healthy individuals with normal blood biochemical values. The plasma coenzyme Q10, malondialdehyde (MDA) and antioxidant enzymes activities (catalase (CAT), superoxide dismutase (SOD), glutathione peroxidase (GPx)) were measured. Subjects with CAD had significant lower plasma coenzyme Q10, CAT and GPx activities and higher MDA and SOD levels compared to those of the control group. The plasma coenzyme Q10 was positively correlated with CAT and GPx activities and negatively correlated with MDA and SOD. However, the correlations were not significant after adjusting for the potential confounders of CAD with the exception of SOD. A higher level of plasma coenzyme Q10 (≥0.52 *μ*mol/L) was significantly associated with reducing the risk of CAD. Our results support the potential cardioprotective impact of coenzyme Q10.

## 1. Introduction

Coenzyme Q10 (also called ubiquinone) is a lipid-soluble benzoquinone with 10 isoprenyl units in the side chain and is a key component of the mitochondrial respiratory chain for adenosine triphosphate (ATP) synthesis [1, 2]. Coenzyme Q10 is an intracellular antioxidant that protects the membrane phospholipids, mitochondrial membrane protein, and low-density lipoprotein-cholesterol (LDL-C) from free radical-induced oxidative damage [3, 4]. Many studies [5–7] have indicated a relationship between low plasma coenzyme Q10 concentration and coronary artery disease (CAD), which may contribute to the higher susceptibility of some individuals to CAD, especially in Asian Indian and Chinese population [8]. However, the relationship between coenzyme Q10 and the prevention of the risk of CAD are controversial. Some studies reported the plasma coenzyme Q10 concentration was not related to the risk of coronary atherosclerosis, and there was no beneficial effect of coenzyme Q10 in patients with CAD [9, 10]. 

Cardiovascular disease (CVD) is the leading cause of death worldwide. The known traditional risk factors for CAD are smoking, obesity, hypertension, a family history of CAD, diabetes mellitus, and hyperlipidemia. In addition to the traditional CAD risk factors, enhanced oxidative stress is a novel risk factor of CAD. Increased oxidative stress is associated with the pathogenesis of CAD [11–14]. Clinical trials have revealed that oxidative stress may increase free oxygen reactive species (ROS) formation and reduce antioxidant defenses [11, 12]. Antioxidant enzymes such as catalase (CAT), superoxide dismutase (SOD), and glutathione peroxidase (GPx) are the first line of defense against ROS, and a decrease in their activities contributes to the oxidant attack on cells, especially in individuals suffering from CAD [15]. As a result, we designed a case-control study to investigate the relationship between coenzyme Q10 concentration and oxidative stress, as well as antioxidant enzymes activities; we also examined the association between coenzyme Q10 and the risk of CAD.

## 2. Materials and Methods

### 2.1. Subjects

The current study was designed as a case-control study. CAD patients were recruited from the cardiology clinic of Taichung Veterans General Hospital in Taiwan. Patients who were identified by cardiac catheterization as having at least 50% stenosis of one major coronary artery or receiving percutaneous transluminal coronary angioplasty (PTCA) were assigned to the case group (*n* = 51). Case subjects with diabetes, liver, renal diseases, or undergoing statin therapy were excluded. None of our subjects had experienced an acute myocardial infarction within the previous 6 months. Control subjects were recruited from the physical examination unit of Taichung Veterans Hospital. Control subjects did not have any illnesses and a history of gastrointestinal disorder, cardiovascular disease (showed normal electrocardiogram), hypertension, hyperlipidemia, liver and renal disease, diabetes, cancer, alcoholism, or other metabolic disease and exhibited normal blood biochemical values, including fasting blood glucose < 6.11 mmol/L, blood urea nitrogen (BUN) < 7.9 mmol/L, creatinine < 123.8 *μ*mol/L, alkaline phosphates < 190 U/L, glutamic oxaloacetic transaminase (GOT) < 35 U/L, and glutamic pyruvate transaminase (GPT) < 45 U/L. Subjects currently taking antioxidant vitamin supplements were also excluded. Informed consent was obtained from each subject. This study was approved by the Institutional Review Board of Taichung Veterans General Hospital in Taiwan. The age, blood pressures, and smoking habits of all subjects were recorded. Blood pressure was measured in each patient after resting for at least 5 min. Body weight, height, waist, and hip circumferences were measured and the body mass index (kg/m^2^) and the waist to hip ratio were then calculated.

### 2.2. Blood Collection and Biochemical Measurement

Fasting venous blood samples (15 mL) were obtained to estimate hematological and vitamin status. Blood specimens were collected in Vacutainer tubes (Becton Dickinson, Rutherford, NJ, USA) with or without containing EDTA as an anticoagulant as needed. Serum and plasma were prepared and then frozen (−80°C) for storage until analysis. Hematological parameters (i.e., serum creatinine, total cholesterol, triacylglycerol, LDL-C, high density lipoprotein-cholesterol (HDL-C)) were measured using an automated biochemical analyzer.

Plasma coenzyme Q10 was measured using high-performance liquid chromatography (HPLC) according to the method of Chu et al. [16] and Littarru et al. [17]. The mean intra- and interassay coefficients of fasting plasma coenzyme Q10 variability were 1.8% and 4.4%, respectively. The mean analytical recovery of plasma coenzyme Q10 was 99.8%. Plasma MDA was determined using the thiobarbituric acid reactive substances (TBARs) method, as described by Botsoglou et al. [18] and Chung et al. [19]. The mean intra- and interassay coefficients of plasma MDA variability were 1.9% and 3.9%, respectively. Red blood cells (RBCs) were diluted with 25x sodium phosphate buffer for SOD and GPx measurements and 250x sodium phosphate buffer for CAT measurement. The methods for measuring CAT, SOD, and GPx in RBCs have previously been described [19] and measurements were performed spectrophotometrically at 240 nm, 325 nm, and 340 nm, respectively. Protein contents of RBCs were determined based on the Biuret reaction of the BCA kit (Thermo, Rockford, IL, USA). The mean intra- and interassay coefficients of protein variability were 0.2% and 3.3%, respectively, in RBCs. The antioxidant enzymes activity levels were expressed as unit/mg of protein. All analyses were performed in duplicate and the variations of repeated determinations were within 10% of the same sample. The analyses of plasma MDA and antioxidant enzymes activities were completed within 7 days.

### 2.3. Statistical Analyses

Data were analyzed using SigmaStat statistical software (version 2.03; Jandel Scientific, San Rafael, CA, USA). The normal distribution of variables was evaluated using the Kolmogorov-Smirnov test. Differences in subjects' demographic data and the hematological measurement data between case and control groups were analyzed using the Student's *t*-test or the Mann-Whitney rank sum test. For categorical response variables, differences between two groups were assessed using the Chi-square test or the Fisher's exact test. To examine the relationships of the plasma coenzyme Q10 concentration and the ratio of coenzyme Q10 to lipid profiles with oxidative stress (MDA) and antioxidant enzymes activities (CAT, SOD, GPx), multiple linear regression analyses were used. We adjusted the potential confounders of CAD, including age, gender, systolic blood pressure, waist to hip ratio, creatinine, and smoking. Adjusted odds ratios (ORs) with 95% confidence intervals (CI) for CAD were calculated from the logistic regression models based on the fourth level (75th percentile) of plasma coenzyme Q10 and the fourth level (75th percentile) of the ratio of coenzyme Q10 to lipid profiles. Data were expressed as means ± standard deviations, and results were considered statistically significant at *P* < 0.05.

## 3. Results

Table 1 shows the demographic data and health characteristics of the subjects. Subjects in the case group had significantly higher values for the number of males, age, systolic blood pressure, body mass index, waist to hip ratio, hematological parameters (i.e., creatinine, LDL-C, TC/HDL-C), and lower HDL-C level than the control group. 

**Figure 1:**
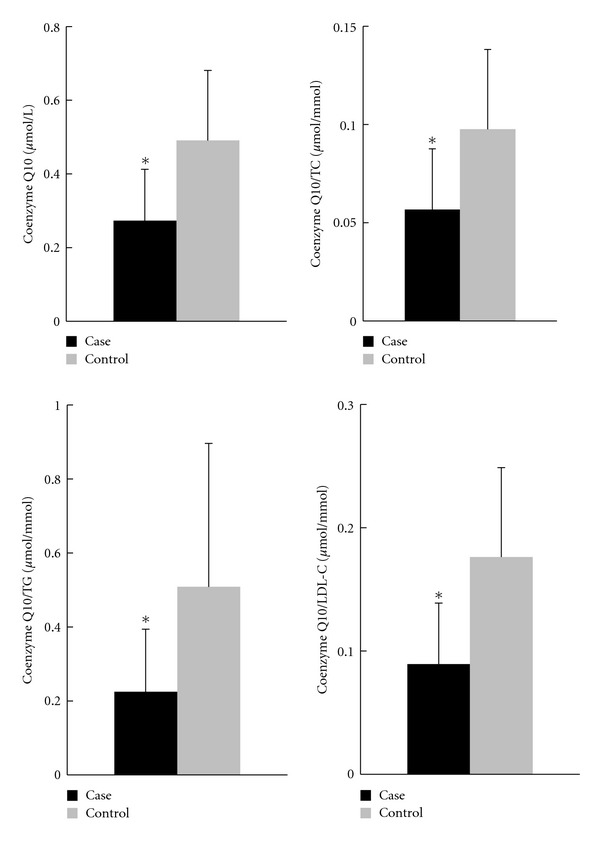
Concentrations of plasma coenzyme Q10 and the ratios of coenzyme Q10 to lipid profiles. *Values were significantly different between case and control groups; *P* < 0.01. LDL-C: low density lipoprotein-cholesterol; TC: total cholesterol; TG: triglyceride.

**Figure 2:**
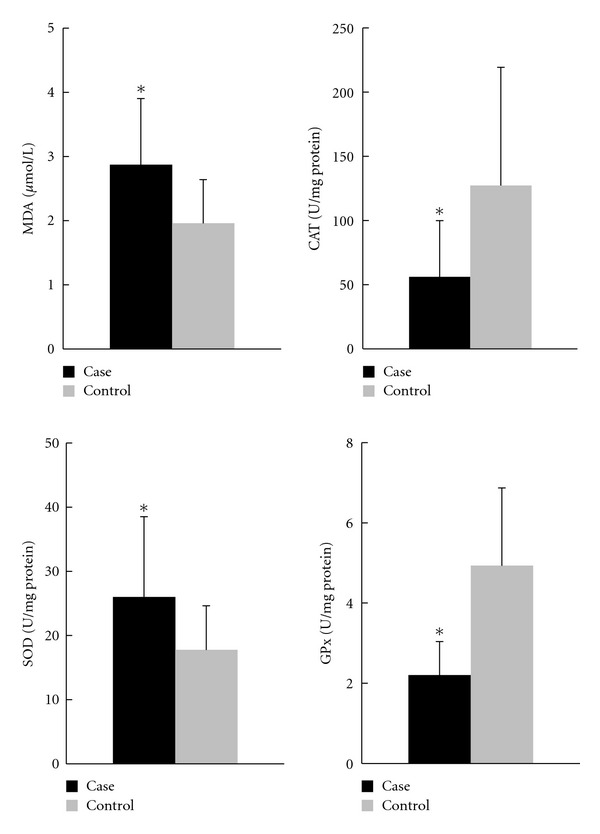
Concentration of lipid peroxidation and antioxidant enzymes activities. *Values were significantly different between case and control groups; *P* < 0.01. CAT: catalase; GPx: glutathione peroxidase; MDA: malondialdehyde; SOD: superoxide dismutase.

 The plasma coenzyme Q10 concentration, lipid peroxidation and antioxidant enzymes activities are shown in Figures 1 and 2. Subjects in the case group had significant decreases in the plasma coenzyme Q10 concentration (*P* < 0.01) and the ratio of coenzyme Q10 to lipid profiles (*P* < 0.01). The value of MDA was significant higher in the case group (*P* < 0.01). With regard to the levels of antioxidant enzymes, subjects in the case group had significant lower CAT (*P* < 0.01) and GPx activities (*P* < 0.01) but higher SOD activities (*P* < 0.01) than the control group. 

The correlations between coenzyme Q10 concentration, lipid peroxidation, and antioxidant enzymes activities are shown in Table 2. The MDA level was significantly negative correlated with the plasma coenzyme Q10 concentration (*β* = −0.72, *P* < 0.05) and the ratio of coenzyme Q10, but the statistical significance were disappeared after adjusting for age and gender or the potential confounders of CAD. The plasma coenzyme Q10 concentration and the ratio of coenzyme Q10 to lipid profiles were significantly positively correlated with CAT and GPx activities but significantly negatively correlated with SOD activities. However, the correlations were not significant after adjusting for the potential confounders of CAD with the exception of SOD. 

Furthermore, we calculated the ORs of CAD based on the fourth level (75th percentile) of plasma coenzyme Q10 concentration and the fourth level (75th percentile) of the ratio of coenzyme Q10 to lipid profiles (Table 3). Subjects with higher plasma coenzyme Q10 (≥0.52 *μ*mol/L) or with a higher ratio of coenzyme Q10 to lipid profiles (coenzyme Q10/TC ≥ 0.10 *μ*mol/mmol, coenzyme Q10/TG ≥ 0.52 *μ*mol/mmol and coenzyme Q10/LDL-C ≥ 0.18 *μ*mol/mmol) had significant reductions in the risk of CAD. 

## 4. Discussion and Conclusion 

The present study showed the plasma coenzyme Q10 concentration had statistically significant reductions in the risk CAD. In Table 3, subjects with a higher coenzyme Q10 concentration (≥0.52 *μ*mol/L) and a higher ratio of coenzyme Q10 to lipid profiles (coenzyme Q10/TG ≥ 0.52 *μ*mol/mmol and coenzyme Q10/LDL-C ≥ 0.18 *μ*mol/mmol) showed a significantly lower risk of CAD even after adjusting for age, gender, and the potential confounders of CAD. This result is similar to a cohort study conducted by Molyneux et al. [20], that followed patients for 2.69 years and suggested that the plasma coenzyme Q10 concentration (either 0.68 or 0.73 *μ*mol/L) was an optimal cut-off point to predict the mortality of patients with chronic heart failure. Patients with lower coenzyme Q10 concentration might have compromised mitochondrial function and correlating to the severity of disease [20]. The cut-off point of plasma coenzyme Q10 (0.52 *μ*mol/L) in this study is also similar with the CORONA (Controlled Rosuvastatin Multinational Study in Heart Failure) trial conducted by McMurray et al. [21], the mortality was significantly increased in the lowest level of coenzyme Q10 (0.49 *μ*mol/L) in a univariate analysis but not in a multivariable analysis. The plasma coenzyme Q10 concentration has been shown to be reduced under statin therapy [21, 22], and we therefore excluded patients who were being treated with statin from this study, and we found that the low coenzyme Q10 level could be a significant predictor of increased CAD risk in a multivariable analysis, even after adjustment for the lipid profiles (LDL-C or TC/HDL-C, *P* = 0.01) (data not shown). 

Although our CAD subjects were stable and had no experience of acute myocardial infarction within the previous 6 months, their plasma coenzyme Q10 concentration was significantly lower than that of control subjects (Figure 1) and the reference values (0.46 *μ*mol/L) [23]. Patient suffering from CAD might suffer loss of coenzyme Q10 under higher oxidative stress [11–14]. Subjects in the case group showed a significant higher lipid peroxide (MDA) level than control (Figure 2, *P* < 0.01), which is an indicator of free radical-induced damage during myocardial ischemia [24, 25]. There was a significant negatively correlations between the plasma coenzyme Q10 and MDA levels (Table 2, Model 1), but the statistical significance disappeared after adjusting for the potential confounders of CAD (Table 2, Models 2 and 3). In addition to oxidative stress, we assessed the activities of the major antioxidant enzymes directly involved in the neutralization of ROS. The activities of CAT and GPx were significantly lower in the case group compared to those of the control group (Figure 2). As shown in Table 2, there was a significantly positive relationship between the levels of plasma coenzyme Q10 and CAT or GPx (Model 1), which disappeared after adjusting for age, gender or other potential confounders of CAD (Model 2 and 3). On the other hand, the activities of SOD were significantly higher in the case group and negative correlated with the concentration of plasma coenzyme Q10, even after adjusting for the potential confounders. The role of antioxidant enzymes defense against the ROS is controversial. In CAD patients, SOD activity may increase to protect against lipid peroxidation and against ROS [15, 26]. Coenzyme Q10 may assist SOD in the uptake of superoxide radical to form oxygen and hydrogen peroxide. 

Traditional CAD risk factors such as gender [23] and age [20, 21, 23, 27] may also influence the plasma coenzyme Q10 concentration. In present study, males (*β* = −0.11, *P* < 0.01) and older patients (*β* = −0.01, *P* < 0.01) had significantly lower levels of plasma coenzyme Q10 in the case group compared to those in the control group. Other CAD risk factors such as blood pressure [28], obesity [21, 29], and smoking [30] may also affect coenzyme Q10 concentration. We have examined the correlations between the plasma coenzyme Q10 concentration and blood pressure, waist to hip ratio, or smoking habits (data not shown). There was a significantly negative correlation between the plasma coenzyme Q10 concentration and systolic blood pressure (*β* = −0.00, *P* = 0.01), smoking (*β* = −0.10, *P* = 0.04), and waist to hip ratio (*β* = −0.26, *P* = 0.09). Therefore, we presume that the plasma coenzyme Q10 level was lower in the case group due to oxidative stress and the traditional CAD risk factors. 

Coenzyme Q10 is a lipid-soluble antioxidant, that is, transported by lipids and lipoprotein (especially LDL-C, 58%) in the blood [31]. Therefore, the value of plasma coenzyme Q10 was normalized relative to the lipid profiles, including TC, TG, or LDL-C, to provide accurate assessments of the amount of plasma coenzyme Q10 [32]. After was lipid normalized, the ratios were significantly lower in the case group compared to those in the control group (Figure 1). The correlations between the ratios of coenzyme Q10 to lipid profiles and antioxidant enzymes activities were similar to plasma coenzyme Q10 without lipid normalization (Table 2). In addition, a higher ratio of coenzyme Q10 to TG or LDL-C showed a significant lower value for the risk of CAD (Table 3). Our results support the potential cardioprotective impact of coenzyme Q10. 

Few studies have investigated the relation between plasma coenzyme Q10 and racial difference, especially in Asian population. An observational study was conducted by Hughes et al. [33] reported Indian males had a significantly lower level of plasma coenzyme Q10 than Chinese male and may contribute the higher susceptibility of this ethnic group to coronary heart disease. The racial difference in lifestyle and nutritional patterns may partly explain the different plasma coenzyme Q10 level [33]. In this study, we have assessed the nutrients intake of all subjects base on 24-h recall (data not shown). Because of the insufficient nutrient databases, we cannot assess coenzyme Q10 intake from 24-h dietary recall, but our CAD subjects had significantly lower antioxidants intake (such as vitamins A and E) than the control. An increase in the concentration of coenzyme Q10 may somehow affect the mitochondrial respiratory function [34] and increase the antioxidants activities [35, 36]; as a result, early supplementation should be administrated in cases of deficiency [36]. 

Our study has two limitations. First, the number of participants was small, although we did recruit more subjects than we expected to recruit (sample size calculation: we expected the differences in mean levels of plasma coenzyme Q10 between case and control groups were to be 0.2 ± 0.3 *μ*mol/L, hence the desired power was set at 0.8 to detect a true effect, and *α* = 0.05 with a minimal simple size of 40 participants in each group). Second, this study was the absence of age and gender matched between case and control groups; as a result, we try to limit these biases by adjusting for the potential confounders of CAD in statistical tests. Lager studies are needed to establish the beneficial effect of coenzyme Q10 in CAD patients. 

Patients with CAD were exposed to a higher level of oxidative stress and a lower coenzyme Q10 concentration. Our results indicate a strong correlation between the plasma coenzyme Q10 and reductions in the risk of CAD. It might benefit in administration of coenzyme Q10 to CAD patients, especially those with low coenzyme Q10 level. 

## Figures and Tables

**Table 1:** Characteristics of subjects.

	Case (*n* = 51)	Control (*n* = 102)	*P* values
Male/female (*n*)	47/4	52/50	<0.01
Age (y)	75.2 ± 8.6^1^ (77.0)	49.8 ± 8.3 (50.0)	<0.01
Systolic blood pressures (mm Hg)	133.3 ± 8.6 (130.0)	118.6 ± 8.6 (120.0)	<0.01
Diastolic blood pressure (mm Hg)	74.1 ± 10.0 (70.0)	76.0 ± 8.2 (77.0)	0.06
Body mass index (kg/m^2^)	25.6 ± 3.1 (25.2)	24.4 ± 3.5 (24.1)	0.03
Waist to hip ratio	0.93 ± 0.1 (0.93)	0.85 ± 0.1 (0.87)	<0.01
Creatinine (*μ*mol/L)	114.9 ± 26.5 (106.1)	88.4 ± 26.5 (88.4)	<0.01
TC (mmol/L)	4.9 ± 0.9 (4.7)	5.1 ± 0.9 (5.2)	0.16
TG (mmol/L)	1.5 ± 0.9 (1.3)	1.5± 0.9 (1.4)	0.85
LDL-C (mmol/L)	3.2 ± 0.8 (3.0)	2.9 ± 0.8 (2.9)	<0.05
HDL-C (mmol/L)	1.0 ± 0.2 (1.0)	1.4 ± 0.4 (1.3)	<0.01
TC/HDL-C	5.4 ± 1.6 (5.0)	3.9 ± 1.2 (3.9)	<0.01
Current smoker^2^, *n* (%)	10 (19.6%)	13 (12.7%)	0.38

^1^Mean ±SD (median).

^2^Current smoker: individuals currently smoking one or more cigarettes per day.

HDL-C: high density lipoprotein-cholesterol; LDL-C: low density lipoprotein-cholesterol; TC: total cholesterol; TG: triglyceride.

**Table 2:** Correlations between plasma coenzyme Q10 and the ratios of coenzyme Q10 to lipid profiles, lipid peroxidation, and antioxidant enzyme activities after adjusting for the potential confounders.

	Plasma coenzyme Q10 (*μ*mol/L)	Coenzyme Q10/TC (*μ*mol/mmol)	Coenzyme Q10/TG (*μ*mol/mmol)	Coenzyme Q10/LDL-C (*μ*mol/mmol)
	*β* ^1^ (*P* value)			

MDA (*μ*mol/L)				
Model 1^2^	−0.72 (<0.05)	−3.81 (0.03)	−0.22 (0.30)	−2.16 (0.02)
Model 2^3^	−0.02 (0.96)	−0.69 (0.70)	−0.01 (0.97)	−0.02 (0.99)
Model 3^4^	−0.06 (0.87)	−0.75 (0.68)	0.02 (0.93)	−0.00 (1.00)
CAT (U/mg protein)				
Model 1	99.23 (<0.01)	502.66 (<0.01)	46.72 (0.03)	276.78 (<0.01)
Model 2	54.09 (0.13)	293.29 (0.08)	26.74 (0.19)	137.34 (0.16)
Model 3	44.31 (0.22)	233.31 (0.18)	16.24 (0.47)	115.06 (0.25)
SOD (U/mg protein)				
Model 1	−14.77 (<0.01)	−63.53 (<0.01)	−8.95 (<0.01)	−38.12 (<0.01)
Model 2	−12.51 (<0.01)	−51.66 (0.01)	−7.87 (<0.01)	−32.15 (<0.01)
Model 3	−12.16 (<0.01)	−50.85 (0.01)	−8.31 (<0.01)	−32.20 (<0.01)
GPx (U/mg protein)				
Model 1	2.22 (<0.01)	8.45 (0.04)	0.69 (0.15)	5.02 (0.02)
Model 2	0.08 (0.92)	−1.50 (0.69)	−0.11 (0.79)	−1.89 (0.39)
Model 3	0.03 (0.98)	−1.58 (0.69)	−0.34 (0.47)	−2.04 (0.37)

^1^Regression coefficient (*N* = 153).

^2^None adjusted.

^3^Adjusted for age and gender.

^4^Same as for model 2 and also adjusted for systolic blood pressure, waist to hip ratio, creatinine, and smoking.

CAT: catalase activity; GPx: glutathione peroxidase; LDL-C: low density lipoprotein-cholesterol; MDA: malondialdehyde; SOD: superoxide dismutase; TC: total cholesterol; TG: triglyceride.

**Table 3:** The odds ratios of coronary artery disease based on the concentrations of coenzyme Q10 and the ratios of coenzyme Q10 to the lipid profiles.

	Odds ratio (95% CI)	*P* value
Coenzyme Q10 < 0.52 *μ*mol/L	1.00	—
Coenzyme Q10 ≥ 0.52 *μ*mol/L		
Model 1^1^	0.08 (0.02–0.36)	<0.01
Model 2^2^	0.11 (0.11–0.90)	0.04
Model 3^3^	0.03 (0.00–0.49)	0.01
Coenzyme Q10/TC < 0.10 *μ*mol/mmol	1.00	—
Coenzyme Q10/TC ≥ 0.10 *μ*mol/mmol		
Model 1	0.14 (0.05–0.43))	<0.01
Model 2	0.24 (0.04–1.56)	0.14
Model 3	0.27 (0.04–1.91)	0.19
Coenzyme Q10/TG < 0.52 *μ*mol/mmol	1.00	—
Coenzyme Q10/TG ≥ 0.52 *μ*mol/mmol		
Model 1	0.19 (0.07–0.52)	<0.01
Model 2	0.03 (0.00–0.35)	<0.01
Model 3	0.03 (0.00–0.51)	0.02
Coenzyme Q10/LDL-C < 0.18 *μ*mol/mmol	1.00	—
Coenzyme Q10/LDL-C ≥ 0.18 *μ*mol/mmol		
Model 1	0.10 (0.03–0.35)	<0.01
Model 2	0.05 (0.01–0.47)	<0.01
Model 3	0.06 (0.01–0.62)	0.02

^1^None adjusted.

^2^Adjusted for age and gender.

^3^Same as for model 2 and also adjusted for systolic blood pressure, waist to hip ratio, creatinine and smoking.

CI: confidence interval; LDL-C: low density lipoprotein-cholesterol; TC: total cholesterol; TG: triglyceride.

## References

[1] Ernster L, Dallner G (1995). Biochemical, physiological and medical aspects of ubiquinone function. *Biochimica et Biophysica Acta*.

[2] Bhagavan HN, Chopra RK (2006). Coenzyme Q10: absorption, tissue uptake, metabolism and pharmacokinetics. *Free Radical Research*.

[3] Alleva R, Tomasetti M, Battino M, Curatola G, Littarru GP, Folkers K (1995). The roles of coenzyme Q10 and vitamin E on the peroxidation of human low density lipoprotein subfractions. *Proceedings of the National Academy of Sciences of the United States of America*.

[4] Singh U, Devaraj S, Jialal I (2007). Coenzyme Q10 supplementation and heart failure. *Nutrition Reviews*.

[5] Littaru GP, Ho L, Folkers K (1972). Deficiency of coenzyme Q 10 in human heart disease. I. *International Journal for Vitamin and Nutrition Research*.

[6] Littarru GP, Ho L, Folkers K (1972). Deficiency of coenzyme Q 10 in human heart disease. II. *International Journal for Vitamin and Nutrition Research*.

[7] Sarter B (2002). Coenzyme Q10 and cardiovascular disease: a review. *The Journal of cardiovascular nursing*.

[8] Hughes K, Lee BL, Feng X, Lee J, Ong CN (2002). Coenzyme Q10 and differences in coronary heart disease risk in Asian Indians and Chinese. *Free Radical Biology and Medicine*.

[9] Van De Vijver LPL, Weber C, Kardinaal AFM, Grobbee DE, Princen HMG, Van Poppel G (1999). Plasma coenzyme Q10 concentrations are not decreased in male patients with coronary atherosclerosis. *Free Radical Research*.

[10] Watson PS, Scalia GM, Galbraith A, Burstow DJ, Bett N, Aroney CN (1999). Lack of effect of coenzyme Q on left ventricular function in patients with congestive heart failure. *Journal of the American College of Cardiology*.

[11] Antoniades C, Tousoulis D, Tentolouris C, Toutouzas P, Stefanadis C (2003). Oxidative stress, antioxidant vitamins, and atherosclerosis. From basic research to clinical practice. *Herz*.

[12] Stocker R, Keaney JF (2004). Role of oxidative modifications in atherosclerosis. *Physiological Reviews*.

[13] Vassalle C, Petrozzi L, Botto N, Andreassi MG, Zucchelli GC (2004). Oxidative stress and its association with coronary artery disease and different atherogenic risk factors. *Journal of Internal Medicine*.

[14] De Pinho RA, De Araújo MC, De Ghisi GLM, Benetti M (2010). Coronary heart disease, physical exercise and oxidative stress. *Arquivos Brasileiros de Cardiologia*.

[15] Gupta S, Sodhi S, Mahajan V (2009). Correlation of antioxidants with lipid peroxidation and lipid profile in patients suffering from coronary artery disease. *Expert Opinion on Therapeutic Targets*.

[16] Chu CS, Kou HS, Lee CJ (2006). Effect of atorvastatin withdrawal on circulating coenzyme Q10 concentration in patients with hypercholesterolemia. *BioFactors*.

[17] Littarru GP, Mosca F, Fattorini D, Bompadre S Method to assay coenzyme Q10 in blood plasma or blood serum.

[18] Botsoglou NA, Fletouris DJ, Papageorgiou GE, Vassilopoulos VN, Mantis AJ, Trakatellis AG (1994). Rapid, sensitive, and specific thiobarbituric acid method for measuring lipid peroxidation in animal tissue, food, and feedstuff samples. *Journal of Agricultural and Food Chemistry*.

[19] Chung Y-C, Chen S-J, Pengspi-Sup H-Y, Chou S-T (2009). Anti hypertensive and antioxidant effects of the Graptopetalum paraguayense E. Walther extract in spontaneously hypertensive rats. *Journal of the Science of Food and Agriculture*.

[20] Molyneux SL, Florkowski CM, George PM (2008). Coenzyme Q10. An independent predictor of mortality in chronic heart failure. *Journal of the American College of Cardiology*.

[21] McMurray JJV, Dunselman P, Wedel H (2010). Coenzyme Q10, rosuvastatin, and clinical outcomes in heart failure: a pre-specified substudy of CORONA (Controlled Rosuvastatin Multinational Study in Heart Failure). *Journal of the American College of Cardiology*.

[22] Molyneux SL, Florkowski CM, Lever M, George PM (2005). Biological variation of coenzyme Q10. *Clinical Chemistry*.

[23] Ghirlanda G, Oradei A, Manto A (1993). Evidence of plasma CoQ10-lowering effect by HMG-CoA reductase inhibitors: a double-blind, placebo-controlled study. *Journal of Clinical Pharmacology*.

[24] Singh RB, Niaz MA, Sharma JP, Kumar R, Bishnoi I, Begom R (1994). Plasma levels of antioxidant vitamins and oxidative stress in patients with acute myocardial infarction. *Acta Cardiologica*.

[25] Singh RB, Wander GS, Rastogi A (1998). Randomized, double-blind placebo-controlled trial of coenzyme Q10 in patients with acute myocardial infarction. *Cardiovascular Drugs and Therapy*.

[26] Bahorun T, Soobrattee MA, Luximon-Ramma V, Aruoma OI (2006). Free radicals and antioxidants in cardiovascular health and disease. *Internet Journal of Medical Update*.

[27] Kalen A, Appelkvist EL, Dallner G (1989). Age-related changes in the lipid compositions of rat and human tissues. *Lipids*.

[28] Rosenfeldt FL, Haas SJ, Krum H (2007). Coenzyme Q10 in the treatment of hypertension: a meta-analysis of the clinical trials. *Journal of Human Hypertension*.

[29] Miles MV, Horn PS, Morrison JA, Tang PH, DeGrauw T, Pesce AJ (2003). Plasma coenzyme Q10 reference intervals, but not redox status, are affected by gender and race in self-reported healthy adults. *Clinica Chimica Acta*.

[30] Gvozdjáková A, Šimko F, Kucharská J, Braunová Z, Pšenek P, Kyselovič J (1999). Captopril increased mitochondrial coenzyme Q10 level, improved respiratory chain function and energy production in the left ventricle in rabbits with smoke mitochondrial cardiomyopathy. *BioFactors*.

[31] Tomasetti M, Alleva R, Solenghi MD, Littarru GP (1999). Distribution of antioxidants among blood components and lipoproteins: significance of lipids/CoQ10 ratio as a possible marker of increased risk for atherosclerosis. *BioFactors*.

[32] Yalcin A, Kilinc E, Sagcan A, Kultursay H (2004). Coenzyme Q10 concentrations in coronary artery disease. *Clinical Biochemistry*.

[33] Hughes K, Lee BL, Feng X, Lee J, Ong CN (2002). Coenzyme Q10 and differences in coronary heart disease risk in Asian Indians and Chinese. *Free Radical Biology and Medicine*.

[34] Estornell E, Fato R, Castelluccio C, Cavazzoni M, Parenti Castelli G, Lenaz G (1992). Saturation kinetics of coenzyme Q in NADH and succinate oxidation in beef heart michondria. *FEBS Letters*.

[35] Lee B-J, Huang Y-C, Chen S-J, Lin P-T (2012). Coenzyme Q10 supplementation reduces oxidative stress and increases antioxidant enzyme activity in patients with coronary artery disease. *Nutrition*.

[36] Singh RB, Wander GS, Rastogi A (1998). Randomized, double-blind placebo-controlled trial of coenzyme Q10 in patients with acute myocardial infarction. *Cardiovascular Drugs and Therapy*.

